# Clinical characteristics and outcomes in those with primary extrahepatic malignancy and malignant ascites

**DOI:** 10.1186/s12876-022-02487-4

**Published:** 2022-09-05

**Authors:** Omar Alshuwaykh, Amanda Cheung, Aparna Goel, Allison Kwong, Renumathy Dhanasekaran, T. Tara Ghaziani, Aijaz Ahmed, Tami Daugherty, Deepti Dronamraju, Radhika Kumari, Mindie Nguyen, W. Ray Kim, Paul Yien Kwo

**Affiliations:** 1 Division of Gastroenterology and Hepatology, Stanford University Medical Center, Stanford, CA USA; 2 Stanford University School of Medicine, 430 Broadway, Pavilion C, 3rd Floor, Redwood City, CA 94063 USA

**Keywords:** Malignant ascites, Peritoneal carcinomatosis, Portal hypertension, Serum ascites albumin gradient

## Abstract

**Background:**

Malignancy-related ascites accounts for approximately 10% of causes of ascites. Our AIM was to characterize the ascites fluid and correlate clinical outcomes in those with extrahepatic malignancy and ascites.

**Methods:**

241 subjects with extrahepatic solid tumors and ascites were reviewed from 1/1/2000 to 12/31/2019, 119 without liver metastasis and 122 with liver metastasis.

**Results:**

Ascites fluid consistent with peritoneal carcinomatosis (PC) was most common, 150/241 (62%), followed by fluid reflecting the presence of portal hypertension (PH), 69/241 (29%). 22/241 (9%) had low SAAG and low ascites fluid total protein, with evidence of PC on cytology and or imaging in 20/22. Lung cancer was the most common malignancy in subjects with ascites due to PC at 36/150 (24%), pancreatic cancer was the most common in subjects with ascites with features of PH at 16/69 (23%). Chemotherapy or immunotherapy alone was the most common management approach. Significantly higher 5-year, 3-year and 1-year mortality rate were noted in subjects with evidence of PC on cytology/imaging versus subjects with no evidence of PC, and in subjects with liver metastasis compared to subjects without liver metastasis. Subjects with pancreatic cancer and evidence of PC on cytology/imaging had higher 1 and 5-year mortality rates compared to subjects without PC.

**Conclusions:**

Ascites in solid tumor malignancy is most commonly due to PC. We also observed ascites fluid with characteristics of PH in 29% of subjects. Higher mortality rates in subjects with peritoneal carcinomatosis and liver metastasis were noted. These findings may help inform prognosis and treatment strategies.

## Background

Among patients with ascites, 85% have evidence of portal hypertension and cirrhosis [1]. Malignancy is the etiology of ascites in approximately 10% of cases [1, 2]. Ascites in the setting of primary extrahepatic solid tumors may occur due to peritoneal seeding, portal hypertension from massive liver metastasis, obstruction of lymphatics, treatment complications, or infiltration of hepatic sinusoids with malignant cells [3, 4]. Peritoneal carcinomatosis appears to cause ascites by producing proteinaceous fluid from tumor cells lining the peritoneum, and extracellular fluid enters the peritoneal cavity to restore the oncotic equilibrium and was reported to account for 53% of malignancy-related ascites in one series [5]. Extrahepatic malignancy with liver metastases may cause ascites due to portal hypertension and is often accompanied by occlusion of hepatic and portal veins by tumor mass effect or tumor thrombosis [5]. Chylous ascites in patients with lymphoma and other malignancies may be caused by lymph node obstruction by tumor and rupture of chyle-containing lymphatics [5]. There may also be multiple etiologies of ascites in the same patient including peritoneal carcinomatosis, hepatic metastasis and cirrhosis with hepatocellular carcinoma [5]. Budd-Chiari syndrome due to an underlying malignancy is another rare etiology accounting for 1% of malignancy related ascites [5]. Multiple chemotherapeutic agents are associated with development of portal hypertension as an idiosyncratic effect including nodular regenerative hyperplasia or sinusoidal obstruction syndrome [6]. To evaluate the etiology of ascites, diagnostic paracentesis for analysis of the ascitic fluid to obtain total protein, albumin, triglyceride, cytology, and other laboratory testing and/cultures is performed. The serum albumin ascites gradient (SAAG) may be calculated by subtracting the ascitic fluid albumin from the serum albumin in simultaneously obtained samples. A serum albumin ascites gradient ≥ 1.1 g/dL with ascitic total protein < 2.5 g/dL is highly suggestive of the presence of portal hypertension, typically caused by liver disease with an accuracy of approximately 97%. A serum albumin ascites gradient ≥ 1.1 g/dL with ascitic total protein of > 2.5 g/dL is highly suggestive of a post hepatic sinusoid source of ascites such as ascites due to right heart failure. A serum albumin ascites gradient < 1.1 g/dL suggests other causes of ascites such as peritoneal carcinomatosis, tuberculosis, and other clinical conditions [7]. Chylous ascites is defined as ascitic fluid triglyceride values > 200 mg/dL [8].

Treatment of malignant ascites has been primarily directed toward the primary tumor combined with peritoneal drainage and the use of diuretics. To date, there are limited reports that classify ascites in this setting and assess response to therapy. The aims of this study were to characterize ascites fluid in those with extrahepatic malignancies and correlate with clinical outcomes in patients with primary extrahepatic solid tumors. Based on these findings, we also propose an algorithm for management of those who present with extrahepatic solid tumor malignancy and ascites.

## Methods

We performed a single-center, electronic database, retrospective analysis of subjects 18 years and older without chronic liver disease who presented with ascites and primary extrahepatic solid tumors including breast cancer, lung cancer, gastric cancer, pancreatic cancer, ovarian cancer, colon cancer, and renal cell cancer between 1/1/2000 and 12/31/2019. Patients with history of any chronic liver disease and evidence of cirrhosis, liver fibrosis, or hepatocellular carcinoma (HCC) were excluded from this study. Subjects were identified via the Stanford Research Repository (STARR) Tools (aka STRIDE-web) of Stanford University electronic data warehouse. Data was fully anonymized after data extraction. Data abstracted included demographic information (age, sex, race), tumor type, serum ascites albumin gradient (SAAG), ascites total protein (TP) level and cytology, abdominal imaging reports, culture results, and ascites treatment regimen. Causes of ascites were categorized as reflecting portal hypertension (PH, SAAG ≥ 1.1 g/dL), with or without elevated total protein that could reflect sinusoidal portal hypertension (SPH, SAAG ≥ 1.1 g/dL and TP < 2.5 g/dL), peritoneal carcinomatosis (PC, SAAG < 1.1 g/dL and TP > 2.5 g/dL), or chylous ascites (ascites fluid triglyceride > 200 mg/dl). Evidence of liver metastases, pericardial disease, and pulmonary hypertension were also captured as was exposure to chemotherapeutic agents known to cause non-cirrhotic portal hypertension. We examined outcomes by the presence or absence of liver metastases and by classification of ascites fluid. Statistical analysis was conducted using RStudio version 1.1.463. Shapiro–Wilk test was used to test normality of continuous variables, two-sample t-test was used to compare normally distributed continues variables, Wilcoxon rank sum test was used to compare continuous variables that were not normally distributed. Categorical variables were compared using Fisher’s exact test. Survival analysis was assessed with Kaplan–Meier method with differences in survival probabilities measured by log-rank testing.

## Results

Two hundred forty-one subjects presented from 1/1/2000 to 12/31/2019 with solid tumors and ascites were reviewed with 119 subjects without liver metastasis and 122 subjects with liver metastasis identified. Table 1 describes baseline characteristics in subjects with malignant ascites and differentiates those with or without liver metastasis. Ascites fluid analysis consistent with peritoneal carcinomatosis (low SAAG, high protein) was most common and observed in 150/241 (62%) subjects. Clinical evidence of peritoneal carcinomatosis was observed on cytology in 96/241 (39.8%) of subjects and imaging in 94/241 (39.0%) of subjects. Among the patients with peritoneal carcinomatosis on cytology or imaging, ascites fluid analysis was consistent with presence of portal hypertension in 46/241 (19%) with 9/241 (4%) having SAAG > 1.1 with low total protein (TP < 2.5 g/dL). Out of the 22 subjects who had low SAAG and low total protein ascites, 20/22 demonstrated peritoneal carcinomatosis on cytology or imaging and the remaining 2/22 had evidence of acute pancreatitis. There was no evidence of nephrotic syndrome, serositis, or tuberculosis in these 22 subjects with low SAAG and low total protein ascites.

**Table 1:** Baseline Characteristics in Subjects with malignancy and ascites stratified by presence of hepatic metastasis

Variable	Without liver metastasis 119 subjects	With liver metastasis 122 subjects	P Value
Portal hypertension*	39/119 (33%)	30/122 (24%)	0.2
Sinusoidal portal hypertension*	4/119 (3%)	9/122 (7%)	0.3
Peritoneal carcinomatosis (PC)*	73/119 (61%)	77/122 (63%)	0.8
Chylous ascites	8/119 (7%)	6/122 (5%)	0.6
SAAG < 1.1 and total protein < 2.5	7/119 (3.5%)	15/122 (12%)	0.1
SAAG < 1.1 and total protein < 2.5 with no evidence of PC on cytology and or imaging	1/119 (0.84%)	1/122 (0.82%)	1
Portal hypertension without PC**	16/119 (13%)	7/122 (6%)	0.04
Sinusoidal portal hypertension without PC**	2/119 (2%)	2/122 (2%)	1
PC based on positive cytology and or imaging	85/119 (71%)	105/122 (86%)	0.007
PC without evidence of PC on cytology or imaging^±^	18/119 (15%)	10/122 (8%)	0.1
Mixed pattern (High SAAG and evidence of PC on cytology and or imaging)	23/119 (19.3%)	23/122 (18.8%)	1
Primary malignancy location			
Breast cancer	12/119 (10%)	29/122 (24%)	0.006
Gastric cancer	24/119 (20%)	9/122 (7%)	0.005
Lung cancer	32/119 (27%)	16/122 (13%)	0.009
Ovarian cancer	15/119 (13%)	5/122 (4%)	0.02
Pancreatic cancer	14/119 (12%)	26/122 (21%)	0.06
Renal cancer	13/119 (11%)	13/122 (11%)	1
Colon cancer	9/119 (8%)	24/122 (20%)	0.008
Treatment regimen			
Diuretics^#^	21/119 (18%)	24/122 (20%)	0.7
Paracentesis^#^	18/119 (15%)	40/122 (33%)	0.001
Peritoneal drain^#^	35/119 (29%)	19/122 (16%)	0.01
Shunt^#^	1/119 (1%)	0/122 (0%)	0.5
Chemotherapy/immunotherapy	44/119 (37%)	39/122 (32%)	0.4

* Subjects with portal hypertension, sinusoidal portal hypertension and peritoneal carcinomatosis based on ascites fluid analysis

** Subjects with PH and SPH based on ascites fluid analysis who had no evidence of PC

on cytology or imaging

^ ± ^Subjects who met criteria for PC based on ascites fluid analysis but did not have demonstrable PC on imaging or positive cytology

^#^ Subjects received these treatments in addition to Chemotherapy/immunotherapy

Ascites fluid analysis consistent with the presence of portal hypertension (high SAAG) in 69/241 (29%), with 13/69 having high SAAG, with low total protein. In this group, 21/69 (30%) subjects in this group had received an agent (azathioprine, oxaliplatin, trastuzumab, or emtansine) that has been associated with development of non-cirrhotic portal hypertension. Ascites fluid analysis consistent with chylous ascites was observed in 14/241 (6%) subjects. Ascites reflecting portal hypertension (SAAG > 1.1) and no evidence of peritoneal carcinomatosis on cytology or imaging was significantly greater in subjects without liver metastasis compared to subjects with liver metastasis (16/119;13% vs. 7/122;2%, *p* = 0.05), with 5 of these 23 subjects receiving either azathioprine, oxaliplatin, trastuzumab, or emtansine. Peritoneal carcinomatosis diagnosis based on cytology or imaging was significantly higher in subjects with liver metastases compared to subjects without liver metastases (105/122;86% vs. 85/119;71%: p < 0.05).

When we examined the 119 individuals without liver metastases, 28 subjects had evidence of pericardial disease (effusion, tamponade, and/or constriction), most of whom had ascites fluid analysis with low SAAG and high total protein (24/28) and the remaining with high SAAG (3/28) or low SAAG and low total protein (1/28). An additional 14 subjects had underlying pulmonary hypertension with ascites fluid analysis showing low SAAG and high total protein in 12/15 subjects and high SAAG in 3/15.

Table 2 shows the distribution of solid tumors in subjects with ascites due to peritoneal carcinomatosis compared to subjects with ascites due to portal hypertension. Lung cancer was the most common malignancy in subjects with ascites due to peritoneal carcinomatosis (24%), and pancreatic cancer was the most common malignancy in subjects with ascites due to portal hypertension (23%). Ovarian cancer was only found in the group with PC 17/150 (11%). Significantly more subjects with renal cell cancer had ascites with features of portal hypertension 13/69 (19%) compared to 11/150 (7%) in the PC group.

**Table 2:** Distribution of solid tumors in subjects with ascites due to peritoneal carcinomatosis and ascites due to portal hypertension based on ascites fluid analysis

Variable	Peritoneal carcinomatosis 150 subjects	Portal hypertension 69 subjects	*P* value
Breast Cancer	23/150 (15%)	14/69 (20%)	0.4
Gastric Cancer	21/150 (14%)	9/69 (13%)	1
Lung Cancer	36/150 (24%)	10/69 (14%)	0.2
Ovarian Cancer	17/150 (11%)	0/69 (0%)	0.002
Pancreatic Cancer	22/150 (15%)	16/69 (23%)	0.1
Renal Cancer	11/150 (7%)	13/69 (19%)	0.02
Colon Cancer	20/150 (13%)	7/69 (10%)	0.7

### Management of ascites

All subjects received chemotherapy/immunotherapy for the underlying malignancy alongside additional therapies for management of ascites (Tables 1, 3, 4). In subjects with peritoneal carcinomatosis, chemotherapy/immunotherapy alone was the most common management approach 52/150 (35%). The remaining patients received additional therapies including paracentesis 35/150 (23%), diuretics 31/150 (21%), and peritoneal drain 31/150 (21%)] and peritoneovenous shunt 1/150 (0.75%) (Table 4). Significantly more subjects with ascites due to peritoneal carcinomatosis and presence of liver metastasis were managed with paracentesis 24/77 (31%) compared to those without liver metastasis 11/73 (15%) (Table 3).

**Table 3:** Comparing management in subjects with and without liver metastasis

Management	Without liver metastasis	With liver metastasis	*P* value
Peritoneal carcinomatosis			
Diuretics	15/73 (20.5%)	16/77 (21%)	1
Paracentesis	11/73 (15%)	24/77 (31%)	0.02
Peritoneal drain	16/73 (22%)	15/77 (19%)	0.8
Shunt	1/73 (1.4%)	0/77(0%)	0.5
Chemotherapy/Immunotherapy*	30/73 (41%)	22/77 (28.5%)	0.1
Portal hypertension			
Diuretics	4/39 (10%)	4/30 (13%)	0.7
Paracentesis	7/39 (18%)	11/30 (37%)	0.1
Peritoneal drain	18/39 (46%)	2/30 (7%)	< 0.001
Shunt	0/39 (0%)	0/30 (0%)	1
Chemotherapy/Immunotherapy*	10/39 (26%)	13/30 (43%)	0.1
Sinusoidal portal hypertension			
Diuretics	0/4 (0%)	0/9 (0%)	1
Paracentesis	1/4 (25%)	3/9 (33%)	1
Peritoneal drain	2/4 (50%)	1/9 (11%)	0.2
Shunt	0/4 (0%)	0/9 (0%)	1
Chemotherapy/Immunotherapy*	1/4 (25%)	5/9 (55.5%)	0.6

*These patients only received chemotherapy or immunotherapy while the others represented in this table received chemotherapy or immunotherapy in addition to the stated therapy

**Table 4:** Comparing management of ascites in subjects with peritoneal carcinomatosis and subjects with portal hypertension

Management	Peritoneal carcinomatosis	Portal hypertension	*P* value
Diuretics	31/150 (21%)	8/69 (11%)	0.13
Paracentesis	35/150 (23%)	18/69 (26%)	0.73
Peritoneal drain	31/150 (21%)	20/69 (29%)	0.23
Shunt	1/150 (0.7%)	0/69 (0%)	1
Chemotherapy/Immunotherapy*	52/150 (35%)	23/69 (33%)	0.88

*These patients only received chemotherapy or immunotherapy while the others represented in this table received chemotherapy or immunotherapy in addition to the stated therapy

In subjects with portal hypertension, chemotherapy or immunotherapy alone was again the most common management approach of ascites 23/69 (33.3%), with additional therapies including peritoneal drain 20/69 (29%), paracentesis 18/69 (26%), and diuretics 8/69 (11.5%) being added. Significantly more subjects with ascites reflecting portal hypertension were managed with peritoneal drain 18/39 (46%) in those without liver metastasis compared to 2/30 (7%) subjects with evidence of liver metastases (Table 3). Diuretics were administered in subjects with portal hypertension and peritoneal carcinomatosis with no significant difference between the groups regardless of liver metastasis (Table 2).

### Outcomes

**Fig. 1:**
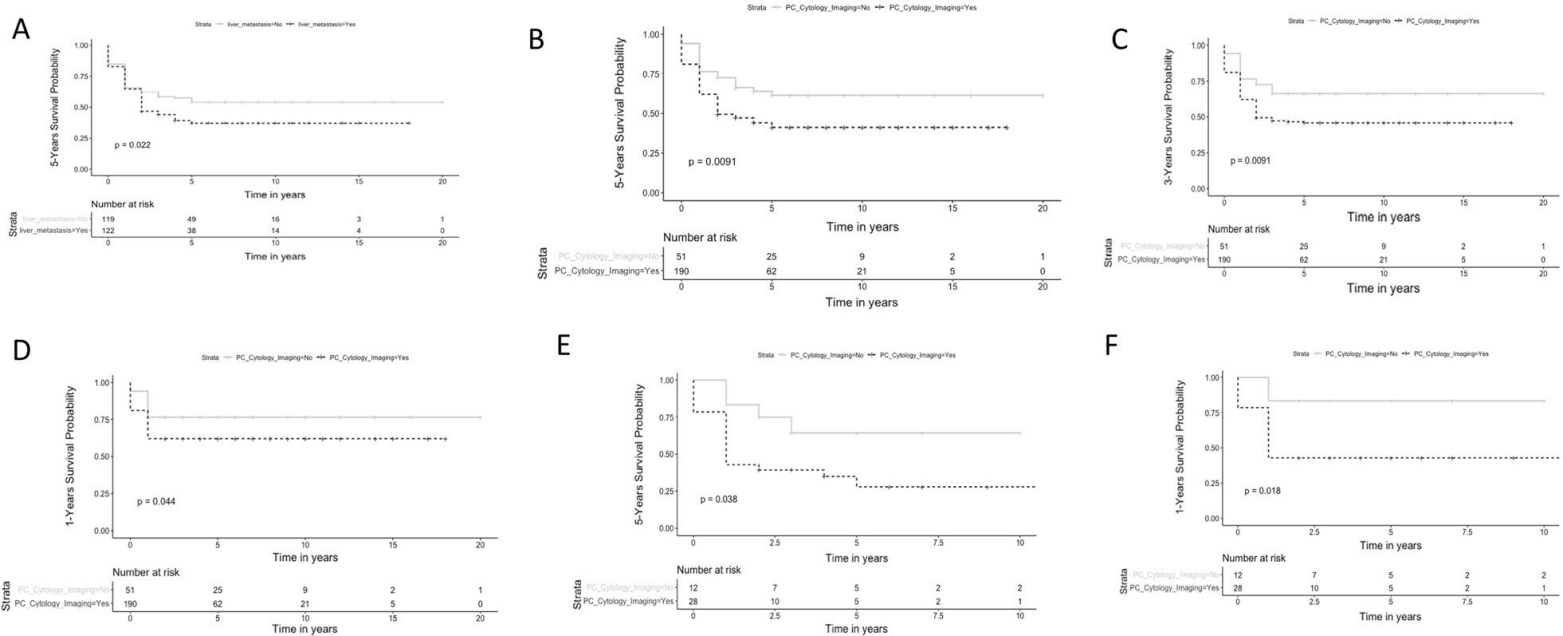
**A** 5-years Kaplan–Meier survival analysis comparing subjects with malignant ascites with and without liver metastasis demonstrating higher mortality rates in those with liver metastasis. **B** 5-years Kaplan–Meier survival analysis comparing subjects with peritoneal carcinomatosis on cytology and or imaging to subjects without evidence of PC. **C** 3-years Kaplan–Meier survival analysis comparing subjects with peritoneal carcinomatosis on cytology and or imaging to subjects without evidence of PC. **D** 1-years Kaplan–Meier survival analysis comparing subjects with peritoneal carcinomatosis on cytology and or imaging to subjects without evidence of PC. **E** 5-years Kaplan–Meier survival analysis comparing subjects with pancreatic cancer and peritoneal carcinomatosis on cytology and or imaging to subjects without evidence of PC. **F** 1-years Kaplan–Meier survival analysis comparing subjects with pancreatic cancer and peritoneal carcinomatosis on cytology and or imaging to subjects without evidence of PC

The 5-year mortality rate in subjects with malignant ascites was significantly higher in subjects with liver metastases compared to subjects without liver metastases 75/122 (61%) vs. 53/119 (45%) (*p* = 0.02) with higher mortality rates noted in those with colon cancer (Table 5, Fig. 1A). Subjects with liver metastasis and ascites reflecting portal hypertension had significantly higher 5-year mortality rate 21/30 (70%) compared to subjects with no liver metastasis and ascites reflecting portal hypertension 14/39 (36%) (Table 5). The 5-year mortality rate in subjects with evidence of peritoneal carcinomatosis on cytology and/or imaging was significantly higher compared to those with no evidence of peritoneal carcinomatoses on cytology or imaging 109/190 (57%) vs. 19/51 (37%), *p* value 0.009 (Fig. 1B). Subjects with evidence of PC on cytology/imaging were also found to have higher 1-year mortality rate 72/190 (38%) vs 12/51 (23%), *p* = 0.04 and 3-year mortality rate 102/190 (54%) vs 17/51 (33%), *p* = 0.009 (Fig. 1C, D). No significant differences were observed in 1-and 3 year mortality rates in subjects with liver metastasis compared to subjects without liver metastasis. In subgroup analysis examining mortality rates by tumor type, subjects with pancreatic cancer and evidence of PC on cytology/imaging had higher 1 and 5-year mortality rates compared to subjects without PC 19/28 (68%) vs. 4/12 (33%), *p* = 0.04 and 16/28 (57%) vs. 2/12 (17%), *p* = 0.02 respectively (Table 6, Fig. 1E, F). No significant differences were observed in 1-year, 3-year, and 5-year mortality rates by tumor type in subjects with liver metastases compared to subjects without liver metastases.

**Table 5:** Comparing 5-year mortality rates in subjects without liver metastasis to subjects with liver metastasis

Variable	Without liver metastasis	With liver metastasis	*P* value
Total 5-year mortality	53/119 (45%)	75/122 (61%)	0.009
Portal HTN	14/39 (36%)	21/30 (70%)	0.007
Sinusoidal portal HTN	2/4 (50%)	7/9 (78%)	0.5
Peritoneal carcinomatosis	34/73 (46.5%)	47/77 (61%)	0.1
CA: Breast cancer	4/12 (33%)	16/29 (55%)	0.3
CA: Gastric cancer	11/24 (46%)	4/9 (44%)	1
CA: Lung cancer	15/32 (47%)	9/16 (56%)	0.8
CA: Ovarian cancer	8/15 (53%)	5/5 (100%)	0.1
CA: Pancreatic cancer	6/14 (43%)	17/26 (65%)	0.2
CA: Renal cancer	7/13 (54%)	8/13 (62%)	1
CA: Colon cancer	2/9 (22%)	16/24 (67%)	0.04

**Table 6:** Subgroup analysis comparing 5-years, 3-years and 1-year mortality rates by cancer type in subjects with evidence of PC on cytology/imaging to subjects without evidence of PC

	PC	No PC	*P* value
5-Years			
Breast	16/31 (52%)	4/10 (40%)	0.63
Gastric	13/30 (43%)	2/3 (67%)	0.4
Lung	20/34 (59%)	4/14 (29%)	0.1
Ovarian	12/19 (63%)	1/1 (100%)	0.78
Pancreatic	19/28 (68%)	4/12 (33%)	0.04*
Renal	7/20 (35%)	2/6 (33%)	0.12
Colon	16/28 (57%)	2/5 (40%)	0.44
3-Years			
Breast	15/31 (48%)	3/10 (30%)	0.43
Gastric	12/30 (40%)	2/3 (67%)	0.33
Lung	20/34 (59%)	4/4 (100%)	0.1
Ovarian	11/19 (58%)	1/1 (100%)	0.78
Pancreatic	17/28 (61%)	4/4 (100%)	0.08
Renal	11/20 (55%)	1/6 (17%)	0.09
Colon	16/28 (57%)	2/5 (40%)	0.44
1-Year			
Breast	6/31 (19%)	3/10 (30%)	0.56
Gastric	10/30 (33%)	2/3 (67%)	0.24
Lung	12/34 (35%)	4/14 (29%)	0.68
Ovarian	10/19 (53%)	0/1 (0%)	0.36
Pancreatic	16/28 (57%)	2/12 (17%)	0.02*
Renal	8/20 (40%)	0/6 (0%)	0.08
Colon	10/28 (36%)	1/5 (20%)	0.47

We also examined mortality rates over 5-year periods to account for potential advances in systemic therapies, but no period effect was noted (data not shown).

## Discussion

This study evaluated and characterized ascites in subjects with primary extrahepatic solid tumors including breast cancer, lung cancer, gastric cancer, pancreatic cancer, ovarian cancer, colon cancer and renal cell cancer. The most common etiology of ascites was peritoneal carcinomatosis based on fluid analysis (62%) or cytology/imaging (79%). Similar to our data, a prior study of 45 patients reported 53% of malignancy-related ascites was due to peritoneal carcinomatosis also across a broad range of malignancies [5]. High SAAG with high total protein accounted for 29% of subjects with malignancy and ascites. Among the patients with high SAAG suggesting noncirrhotic portal hypertension based on ascites fluid analysis, 67% had evidence of peritoneal carcinomatosis on cytology or imaging suggesting that multiple mechanisms are contributing to the development of ascites. Interestingly, 9% had low SAAG and low ascites fluid total protein levels with no evidence of nephrotic syndrome, pancreatic ascites, tuberculous peritonitis, or serositis. The median serum albumin level in this group was low 1.99 g/dl (IQR 1.8–2.03) likely as a reflection of cachexia and other factors and may explain the low SAAG findings.

### Liver metastases versus no liver metastases

In those with ascites and no liver metastases, peritoneal carcinomatosis was the most common etiology of ascites in this group at 61%, and lung cancer was the most common malignancy in this group at 27%. Significantly more subjects without liver metastases and no evidence of peritoneal carcinomatosis on cytology or imaging had peritoneal fluid reflecting portal hypertension with high SAAG, 16/119 (13%) compared to subjects with liver metastasis 7/122 (6%). A portion of these individuals (3/16 and 2/7 respectively) received chemotherapies known to be associated with non-cirrhotic portal hypertension which may in part explain these findings. Chemotherapy/immunotherapy administration alone was the most common approach to manage ascites in the no liver metastases subjects with 37% of subjects receiving this type of therapy.

Among subjects with ascites and liver metastases, peritoneal carcinomatosis was again the most common etiology of ascites in this group at 63%, a rate similar to those without liver metastases, though breast cancer was the most common malignancy in this group at 24% (Table 3). In one malignant ascites series, breast cancer was also reported to be the most common extrahepatic tumor associated with ascites [9]. Peritoneal carcinomatosis was commonly present with liver metastases suggesting that the presence of liver metastases reflects a higher overall disease burden. Drainage of peritoneal fluid by paracentesis or indwelling catheter combined with chemotherapy/immunotherapy was the most common method of managing ascites in these subjects at 33% suggesting that fluid accumulation may be more difficult to control.

We found that the 5-year mortality rate in subjects with liver metastases was significantly higher at 61% compared to 45% in subjects without liver metastases regardless of ascites characteristics with no differences when examined in 5-year period increments (Fig. 1a). The presence of liver metastases was reported to be an independent predictor for mortality in patients with solid tumors who developed tumor lysis syndrome [10]. Similar to our findings, one prior report has noted that the presence of liver metastasis in subjects with malignant ascites was an independent prognostic factor associated with poor outcomes [11]

### Ascites reflecting peritoneal carcinomatosis or portal hypertension

Peritoneal carcinomatosis was the most common etiology of ascites in our cohort with lung cancer being the most common malignancy in subjects with ascites due to PC at 24%. We noted that patients with ovarian cancer had ascites consistent with peritoneal carcinomatosis only. To date, no specific solid tumor has been associated with a higher rate of peritoneal carcinomatosis. In the entire cohort, systemic chemotherapy or immunotherapy alone was the most common management approach of ascites in subjects with peritoneal carcinomatosis 52/150 (35%). The most common approach in those with peritoneal carcinomatosis and no liver metastases (41%) was to administer chemotherapy/immunotherapy alone, whereas the majority of subjects with peritoneal carcinomatosis and liver metastases (31%) required paracentesis in addition chemotherapy/immunotherapy indicating multiple therapies may be required in the setting of malignancy and hepatic metastases to control the ascites. We noted a higher 1 and 3-year in those with ascites due to peritoneal carcinomatosis (Fig. 1B–D). In Subgroup analysis examining mortality rates by tumor type, subjects with pancreatic cancer and evidence of PC on cytology/imaging had higher 5-years and 1-year mortality rates compared to subjects without PC (Fig. 1E, F). A prior study also reported poor prognosis in subjects with pancreatic cancer and peritoneal carcinomatosis [12].

Low SAAG ascites has been reported to be caused by peritoneal carcinomatosis in addition to tuberculous peritonitis, nephrotic syndrome, and pancreatitis [1, 13]. We identified 20/241 (8%) subjects with low SAAG and low total protein, and all had evidence of peritoneal carcinomatosis on cytology and or imaging. Low SAAG ascites has been reported in up to 20% of those with malignant ascites in prior reports and in our cohort, the serum albumin levels were low in our cohort (median 1.99, IQR 1.83–2.13) [14]. In our cohort, the diagnostic accuracy of high ascites fluid total protein in diagnosing PC was 75%, similar to prior studies [15].

Interestingly, evidence of portal hypertension was present in 33% of subjects with no liver metastases and 24% of subjects with liver metastases with pancreatic cancer being the most common malignancy in subjects with ascites due to portal hypertension at 23%. Significantly more subjects with renal cell cancer had high SAAG ascites (13/69; 19%) compared to (11/150; 7%) in the PC group with just 3/13 subjects receiving azathioprine, oxaliplatin, trastuzumab, or emtansine, all therapies associated with non-cirrhotic portal hypertension. In our retrospective series, chemotherapy or immunotherapy alone was the most common management approach for the ascites in subjects with evidence of portal hypertension 23/69 (33.3%). No liver biopsies were performed in the subjects without liver metastases group to determine if sinusoidal infiltration of tumor, nodular regenerative hyperplasia or other etiology was present nor were hepatic venous pressure measurements performed. We noted limited use of diuretic therapies in those with high SAAG ascites (11%), though diuretics should be a first line consideration in this subgroup [16]. There are limitations to our study. We reported a single center and retrospective data without a standardized approach. Not all diagnostic tests were done in all subjects. No liver biopsies or pressure measurements were performed to explain or confirm the findings of portal hypertension.

In summary, although ascites in solid tumor malignancy is most commonly due to peritoneal carcinomatosis, we observed characteristics of portal hypertension with SAAG > 1.1 in 29% of subjects. We also observed that the presence of liver metastasis was not the sole contributor to the development of ascites with features of portal hypertension which could be related to prior chemotherapies or other factors. With ascites fluid analysis, we believe that ascites in the setting of extrahepatic malignancy can be managed with a combination of therapies directed toward the tumor, especially in the era of increasing targeted therapies, and if present portal hypertension [17]. We also noted that despite advances in targeted chemo/immune-oncology therapies and the decreased mortality rates in cancer patients, patients with evidence of PC continues to have high mortality rates. Based on our results, we propose for the management of ascites in subjects with primary extrahepatic solid tumors, we propose that these subjects undergo diagnostic paracentesis to obtain cytology, albumin, and total protein. If the SAAG is ≥ 1.1 with/without TP < 2.5, then treatment of underlying tumor and initiation of diuretic therapy (furosemide/spironolactone at standard doses) is warranted. If these measures fail to control the ascites, then initiation of serial paracenteses should be the next step while adjusting the therapy of the underlying malignancy. If ascites still remains problematic, placement of peritoneal drain/shunt, should be considered along with a discussion of goals of care. If the SAAG ≥ 1.1 with TP > 2.5, then treatment of underlying tumor and initiation of paracentesis should be the initial management steps. If these measures fail to control the ascites, then placement of a peritoneal drain/shunt, adjustment of therapy toward the primary extrahepatic malignancy if required, again with a discussion of goals of care should be considered. Finally, we noted multiple presentations that predicted higher mortality including those with liver metastasis or peritoneal carcinomatosis on cytology and or imaging.

## Conclusions

Ascites in solid tumor malignancy is most commonly due to peritoneal carciniomatosis. We also observed ascites fluid with characteristics of portal hypertension in 29% of subjects. We observed higher mortality rates in subjects with peritoneal carcinomatosis and liver metastasis. These findings may help inform prognosis and treatment strategies.

## Data Availability

he datasets during and/or analysed during the current study available from the corresponding author on reasonable request.

## Abbreviations

SAAG: Serum albumin ascites gradient
PC: Peritoneal carcinomatosis
HCC: Hepatocellular carcinoma
TP: Total protein
PH: Portal hypertension
SPH: Sinusoidal portal hypertension
